# Long non-coding RNA CTA sensitizes osteosarcoma cells to doxorubicin through inhibition of autophagy

**DOI:** 10.18632/oncotarget.16356

**Published:** 2017-03-18

**Authors:** Zhengguang Wang, Zhendong Liu, Song Wu

**Affiliations:** ^1^ Department of Orthopaedics, The Third Xiangya Hospital, Central South University, Changsha, Hunan 410013, China

**Keywords:** osteosarcoma, long non-coding RNA, doxorubicin, resistance, autophagy

## Abstract

Recently, several long non-coding RNAs (lncRNAs) have been implicated in osteosarcoma (OS). However, the regulatory roles of lncRNAs in chemotherapy resistance of OS still remain unclear. This study aimed to screen a novel lncRNA that contributes to chemotherapeutic resistance of OS, and to explore the underlying mechanisms. Our data showed that lncRNA CTA was markedly downregulated in OS tissues compared to their matched non-tumor tissues, and low expression of lncRNA CTA was significantly associated with the advanced clinical stage and tumor size. In addition, OS patients with low lncRNA CTA levels showed a worse prognosis when compared with those with high expression of lncRNA CTA. Furthermore, we report that lncRNA CTA has an inverse relationship with miR-210 expression in OS tissues. LncRNA CTA could be activated by doxorubicin (DOX), and could promote OS cell apoptosis by competitively binding miR-210, while inhibit cell autophagy. On the other hand, lncRNA CTA was downregulated in DOX-resistant OS cells. Overexpression of lncRNA CTA reduced autophagy and subsequently overcame DOX resistance of OS *in vitro* and *in vivo*. Therefore, we demonstrate that lncRNA CTA is an essential regulator in DOX-induced OS cell apoptosis, and the lncRNA CTA-miR-210 axis plays an important role in reducing OS chemoresistance.

## INTRODUCTION

Osteosarcoma (OS) is the most common cancer in bone with poor prognosis, mainly due to chemotherapy resistance [1]. Effective therapeutic strategy is urgently needed, and the molecular mechanism of osteosarcoma should be firstly clear.

Emerging evidence have revealed that non-coding RNAs including long non-coding RNA (lncRNA) and microRNAs (miRs) are involved in drug resistance [2, 3]. MiRs generally lead to mRNA degradation or inhibition of translation via directly binding to 3′-untranslated regions (3′-UTR) of their target genes mRNA [4–6]. Various miRs have been found to may become potential therapeutic targets or candidates for osteosarcoma [7]. MiR-210 is an important miR in cancer development [8, 9]. Dysregulation of miR-210 directly modulates changes in mRNA transcription associated with aberrant regulation of cell morphology and metastasis [10]. For example, miR-210 overexpressed non-small cell lung carcinoma cells have reduced caspase-3 activity, resulting in decreased apoptosis, and also able to proliferate in a gamma-irradiated environment [11, 12]. Increased levels of miR-210 expression also are associated with poor cancer patient outcomes, including pancreatic ductal adenocarcinoma [13], glioma [14], and breast cancer [15]. And miR-210 upregulation shows a strong correlation with tumor aggressive progression of pediatric osteosarcoma [16]. Thus, miR-210 acts as an oncogene in many types of cancers, and has been the most extensively studied.

LncRNAs are defined as endogenous cellular RNAs more than 200 nucleotides in length [17]. In recent years, several lncRNAs have been shown to be involved in the cancer progression [18, 19]. For example, Zhao H et al showed that lncRNA HNF1A-AS1 promoted the progression of OS via regulating the activity of the Wnt/β-catenin pathway [20]. Li W et al suggested that lncRNA-UCA1 may contribute to osteosarcoma initiation and progression, and may be a novel prognostic marker [21]. Wang Y et al demonstrated that lncRNA LINC00161-miR-645-IFIT2 signaling axis played an important role in chemoresistance of osteosarcoma [22].

These studies indicate that miRs and its target lncRNAs may play an important role in osteosarcoma cell proliferation, apoptosis and chemotherapy resistance. However, the relationship between miR-210 and lncRNAs in chemotherapy resistance of osteosarcoma cells remain unknown. Thus, the aim of this study was to screen a novel lncRNA that contributed to chemotherapeutic resistance of OS and identify the underlying regulatory mechanisms.

## RESULTS

### The expression of miR-210 related lncRNAs in osteosarcoma tissues

**Figure 1:**
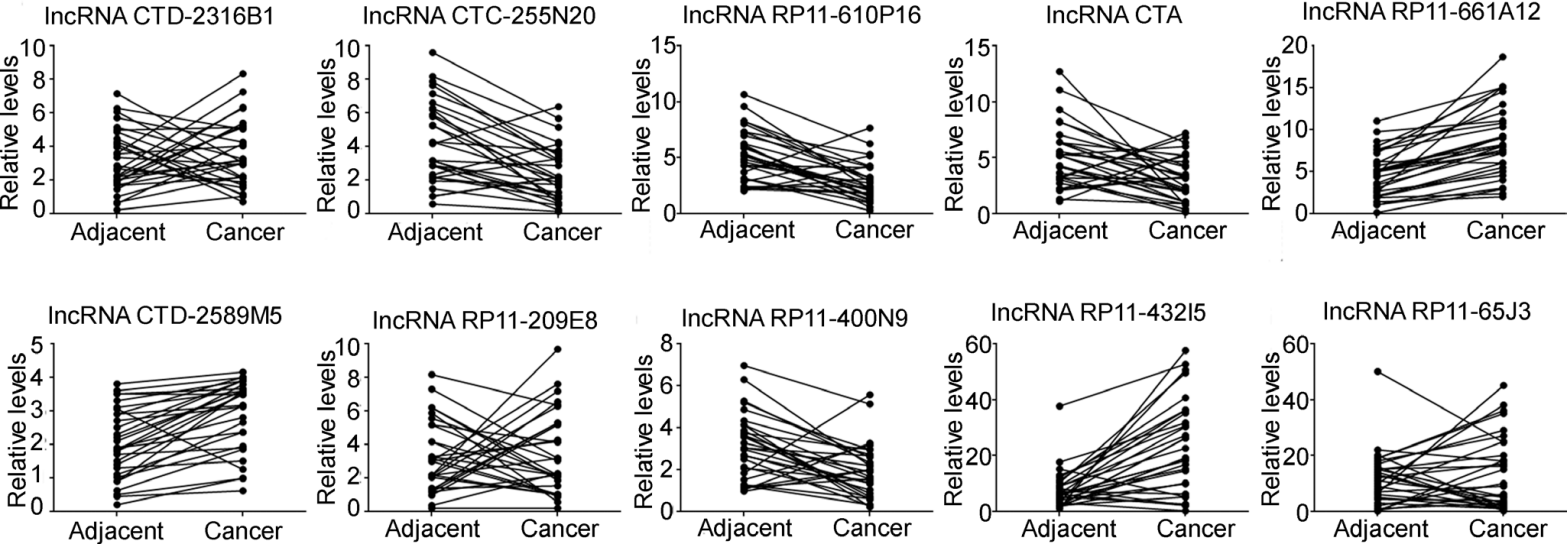
The expression of miR-210 related lncRNAs in osteosarcoma tissues qPCR was performed to analyze the expression of ten lncRNAs that were associated with miR-210 in osteosarcoma tissues and the match adjacent tissues. The expression of lncRNA CTC-255N20, lncRNA RP11-610P16, lncRNA CTA and lncRNA RP11-432I5 in osteosarcoma tissues was significantly higher than in the matched adjacent tissues.

**Figure 2:**
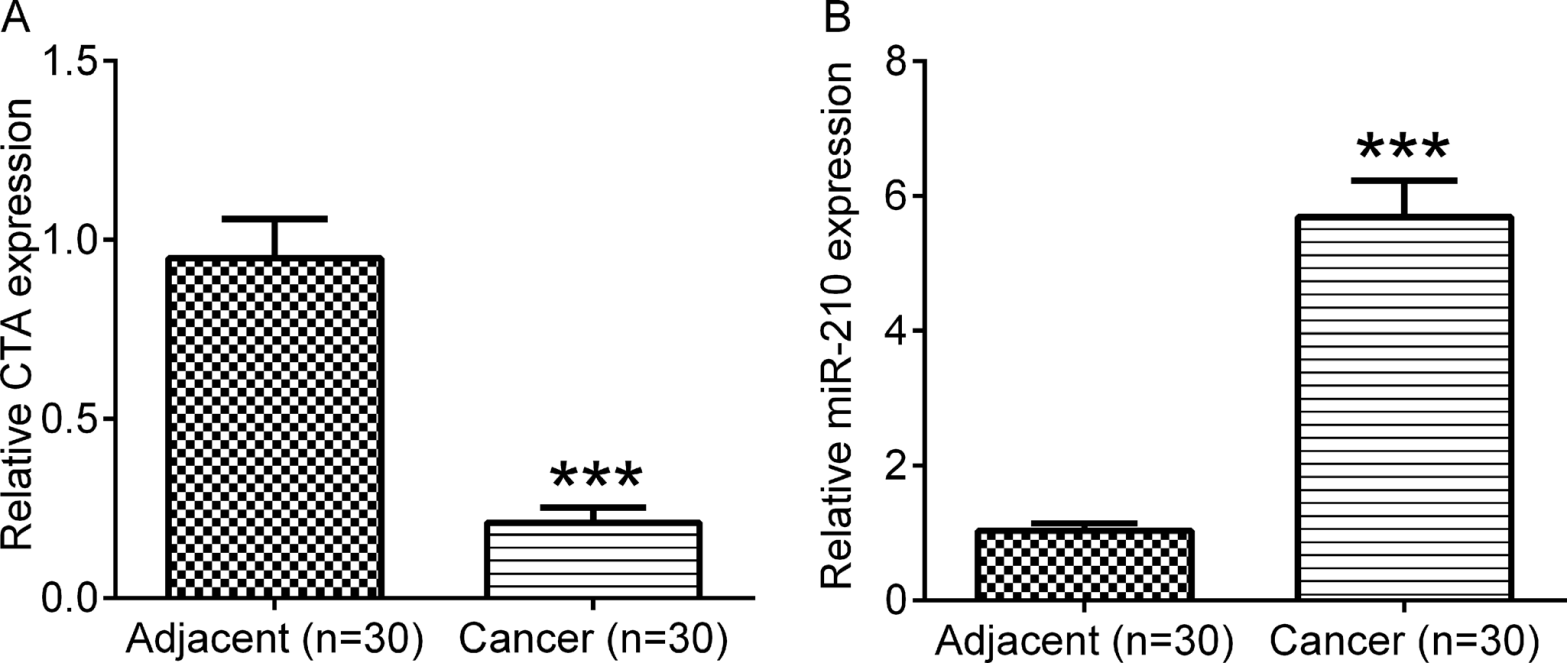
The expression of CTA and miR-210 in OS tissues (**A**) Real-time RT-PCR was performed to determine the relative CTA level in osteosarcoma and adjacent tissues. (**B**) Real-time RT-PCR was performed to determine the relative miR-210 level in osteosarcoma and adjacent tissues. ***P < 0.001.

**Figure 3:**
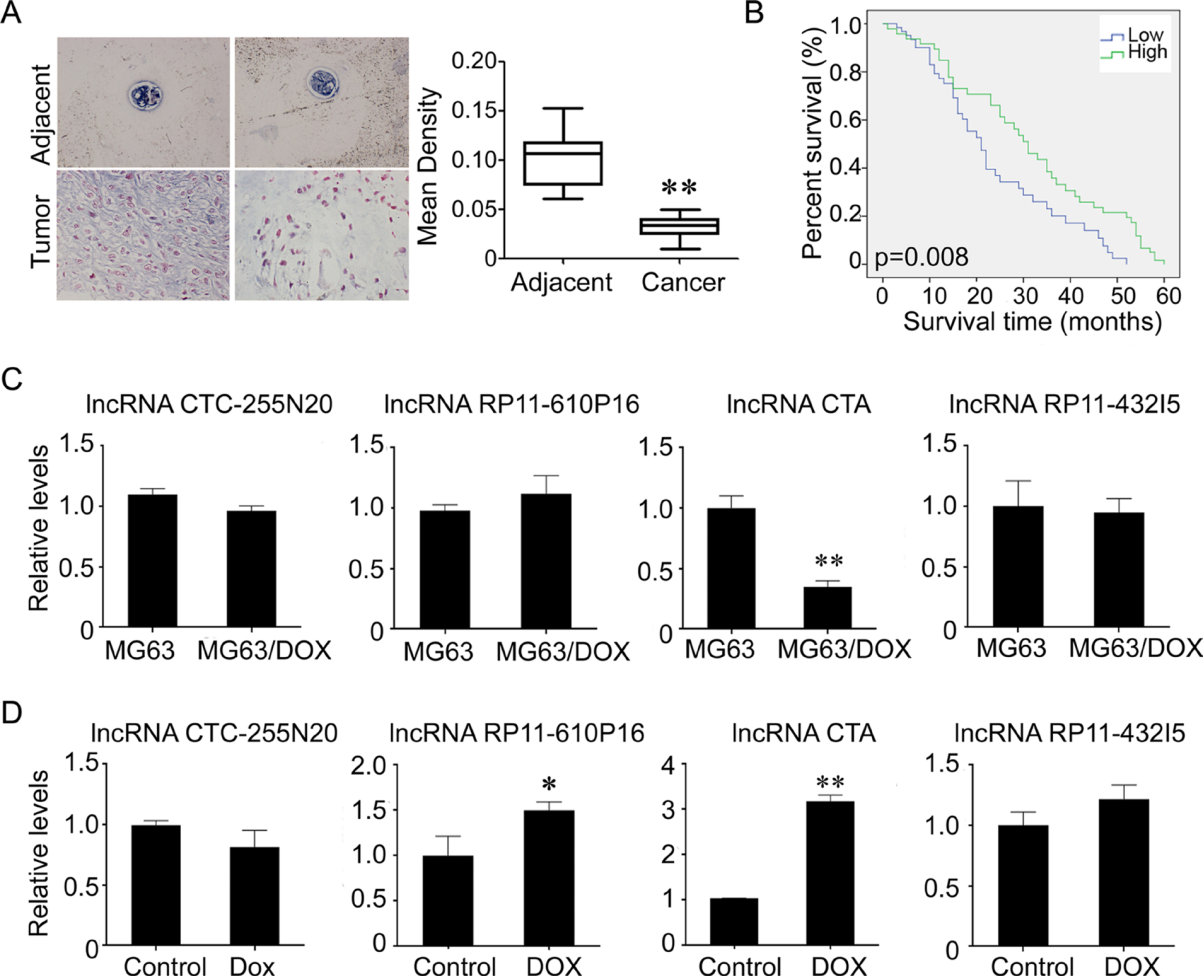
Low lncRNA CTA predicts a poor prognosis in patients with osteosarcoma, and is associated with DOX-resistance in MG-63 cells (**A**) ISH was performed to analyze the expression of lncRNA CTA in osteosarcoma tissue microarray (Left. cancer tissue, *n* = 92; adjacent tissue, *n* = 12), and quantification (Right). The positive signals for lncRNA CTA were stained in blue violet in osteosarcoma and normal osteoblast cells, while the bone matrix around the osteoblast is not colored. (**B**) Kaplan-Meier postoperative survival curve for patterns of patients with osteosarcoma and lncRNA CTA expression. Osteosarcoma patients with low lncRNA CTA expression showed a shorter survival time than those with high lncRNA CTA expression. (**C**) qPCR was performed to analyze the expression of four lncRNAs that were decreased in osteosarcoma tissues in DOX-resistant MG-63 cells and the parental cells. (**D**) qPCR was performed to analyze the expression of four lncRNAs that were decreased in osteosarcoma tissues in MG-63 cells stimulated with DOX. *P < 0.05, **P < 0.01.

MiR-210 is an important gene on regulation of cell proliferation, cell apoptosis and consequent development of many types cancer, including osteosarcoma. In the present study, to investigate role of miR-210 related lncRNAs on progression of osteosarcoma, we used the bioinformatic tool (http://www.mircode.org/) to screen the candidate lncRNAs. We found that there were many lncRNAs could interact with miR-210, and we selected ten candidate lncRNAs with conserved interaction to miR-210. We firstly compared the expression levels of the ten lncRNAs (ENSG00000249362.1, lncRNA CTD-2316B1; ENSG00000250343.1, lncRNA CTC-255N20; ENSG00000251611.1, lncRNA RP11-610P16; ENSG00000253603.1, lncRNA CTA; ENSG00000254859.1, lncRNA RP11-661A12; ENSG00000255007.1, lncRNA CTD-2589M5; ENSG00000259237.1, lncRNA RP11-209E8; ENSG00000259793.1, lncRNA RP11-400N9; ENSG00000260612.1, lncRNA RP11-432I5; ENSG00000261334.1, lncRNA RP11-65J3) in osteosarcoma tissues (*n* = 30) and their matched adjacent non-tumor tissues. Total RNA was isolated from the tissues, and real-time RT-PCR was performed to examine the expression levels of these lncRNAs. Our data showed that the expression of lncRNA CTC-255N20, lncRNA RP11-610P16, lncRNA RP11-400N9 and lncRNA CTA were markedly decreased in osteosarcoma tissues, whereas the levels of lncRNA RP11-661A12, lncRNA CTD-2589M5 and lncRNA RP11-432I5 were significantly increased in osteosarcoma tissues compared to their matched non-tumor tissues (Figure 1). And there were no significant difference in the expression of lncRNA CTD-2316B1, lncRNA RP11-209E8 and lncRNA RP11-65J3 in osteosarcoma tissues and their matched non-tumor tissues (Figure 1). Our results also showed that CTA expression was significantly decreased in tumor tissues compared with adjacent tissues (revised Figure 2A), whereas the miR-210 expression was significantly upregulated in tumor tissues compared with adjacent tissues (revised Figure 2B). Considering the negatively regulatory function of miR-210 on its target genes, we selected the lncRNAs that downregulated in osteosarcoma tissues to further analysis. To investigate the role of these lncRNAs on doxorubicin (DOX) resistant osteosarcoma cells, MG63/DOX, we detected their expression in these cell lines. We found that chronic exposure to DOX induces significant downregulation of CTA in osteosarcoma MG63/DOX cells compared with their parental cells (Figure 3C), while there were no significant differences on the expression of lncRNA CTC-255N20, lncRNA RP11-610P16 and lncRNA RP11-432I5 (Figure 3C). In addition, we further examined their expression in osteosarcoma MG63 cells treated with DOX. As shown in Figure 3D, real-time RT-PCR data showed that the expression level of lncRNA CTA were also induced by DOX in osteosarcoma cell lines when compared with control cells, indicating lncRNA CTA had a close relationship on drug resistance in osteosarcoma.

### Low level of lncRNA CTA predicates poor prognosis of osteosarcoma patients

To further reveal the role of lncRNA CTA in osteosarcoma, we evaluated the association between its expression and the clinicopathological characteristics of osteosarcoma in tissue microarray containing 40 cases of osteosarcoma and 12 cases of normal bone tissues. As shown in Figure 3A, the results of ISH showed that the expression of lncRNA CTA osteosarcoma tissues was significantly downregulated compared with those of in normal bone tissues. And then 92 patients were classified into two groups: the patients with low lncRNA CTA expression (score < 1) and the patients with high lncRNA CTA expression (score ≥ 1). As indicated in Table 1, low expression of lncRNA CTA was significantly associated with the advanced clinical stage and tumor size. However, we found no association between the lncRNA CTA expression and the age and gender (Table 2). These findings suggest that the decreased expression of lncRNA CTA is associated with the malignant progression of osteosarcoma. In addition, we further analyzed the relationship between the lncRNA CTA expression and the survival time of osteosarcoma patients. Our data showed that osteosarcoma patients with low lncRNA CTA expression showed a worse prognosis when compared with those with high level of lncRNA CTA (Figure 3B). Accordingly, our data demonstrates that low expression of lncRNA CTA can predicate a poor prognosis of patients with osteosarcoma.

**Table 1:** Association between lncRNA CTA expression and clinical features of patients with osteosarcoma

Clinicopathological features	Group	Total	CTA expression		χ^2^ test *P* value
			Low	High	
**Age (years)**	> 25	40	21	19	0.307
	≤ 25	52	22	30	
**Gender**	Female	35	12	23	0.797
	Male	57	31	26	
**Clinical stage**	I	39	11	28	0.001**
	II	53	32	21	
**Tumor size (cm)**	8	41	3	38	0.002**
	≥ 8	51	40	11	

**Table 2:** The sequences of primers used in PCR

Genes	Primers 5′-3′
H-ENSG250343.1-F	GTCCTGGCTGTGGGTCTA
H-ENSG250343.1-R	CATCCCTATTACTGCTCCTAT
H-ENSG255007.1-F	GAGTCTAGTCCCTGATGGTC
H-ENSG255007.1-R	GCACATTTAATTTCGCTCT
H-ENSG259237.1-F	TTCTGCTCTTCTGCCCTGTG
H-ENSG259237.1-R	GTGCGATTCTATCTCGTTCA
H-ENSG260612.1-F	AAAATCCATCTGCCCTCA
H-ENSG260612.1-R	TTCCCTTCTCCAGACTTCC
H-ENSG261334.1-F	CTGGGCTAGGGAGATTGGAG
H-ENSG261334.1-R	GGAAGTAGAACGCCTTGACACT
H-ENSG259793.1-F	GCTGTTGCCTACACCTGTT
H-ENSG259793.1-R	CCACGGTTTCTGCTTCTT
H-ENSG249362.1-F	CTTGACAAAAGCACATCC
H-ENSG249362.1-R	TCAGTGGGGTAAGCAGTA
H-ENSG251611.1-F	CTTCCTTTAGCTGTCTACCACC
H-ENSG251611.1-R	AGGAGCCACTTCTTGACTGAG
H-ENSG253603.1-F	CGACACGGAAACCTGAGAA
H-ENSG253603.1-R	CAGAAGGACGAGAAGCCAAG
H-ENSG254859.1-F	CCCGCCAAGAAAGGCAGAT
H-ENSG254859.1-R	CGACCATACCCAATAGAAGAGC
H-GAPDH-F	TCTGATTTGGTCGTATTGGG
H-GAPDH-R	TGGAAGATGGTGATGGGATT

### LncRNA CTA sensitizes osteosarcoma cells to DOX via inhibition of autophagy

**Figure 4:**
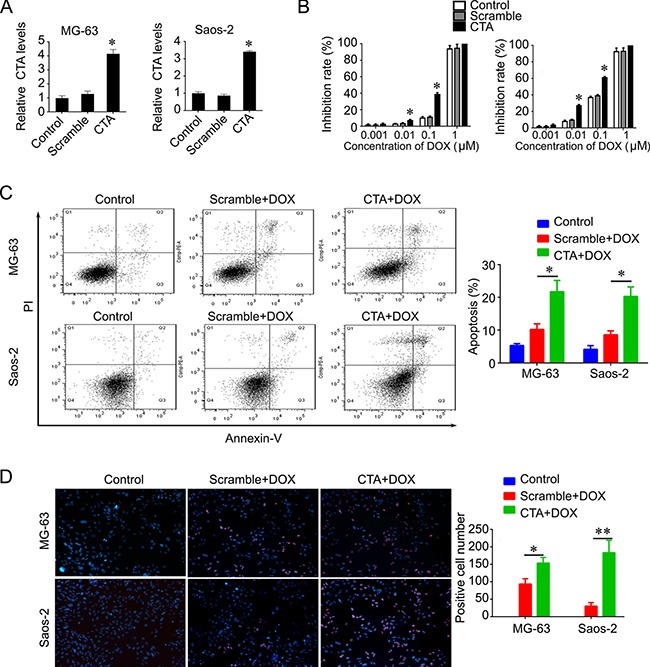
LncRNA CTA sensitizes osteosarcoma cells to DOX (**A**) LncRNA CTA was overexpressed in MG-63 and Saos-2 cells. (**B**) MTT was performed to measure the inhibition rate after indicated treatment. Upregulation of lncRNA CTA significantly increased the inhibition rate induced by DOX. (**C**) Flow cytometry was performed to measure the apoptosis rate after indicated treatment. Upregulation of lncRNA CTA significantly enhanced the apoptosis rate induced by DOX. (**D**) TUNEL was performed to measure the apoptotic cells after indicated treatment (left), and quantification (right). Upregulation of lncRNA CTA significantly increased the number of apoptotic cells induced by DOX. All the experiments were independently performed three times. Data were presented as mean ± standard deviation.*P < 0.05, **P < 0.01.

We further investigated whether lncRNA CTA had a synergic effect with DOX on the proliferation and apoptosis of osteosarcoma cells. We found that overexpression of lncRNA CTA markedly enhanced DOX-induced apoptosis and inhibition of proliferation in osteosarcoma cells, MG63 and Saos-2 cells (Figure 4A–4D).

**Figure 5:**
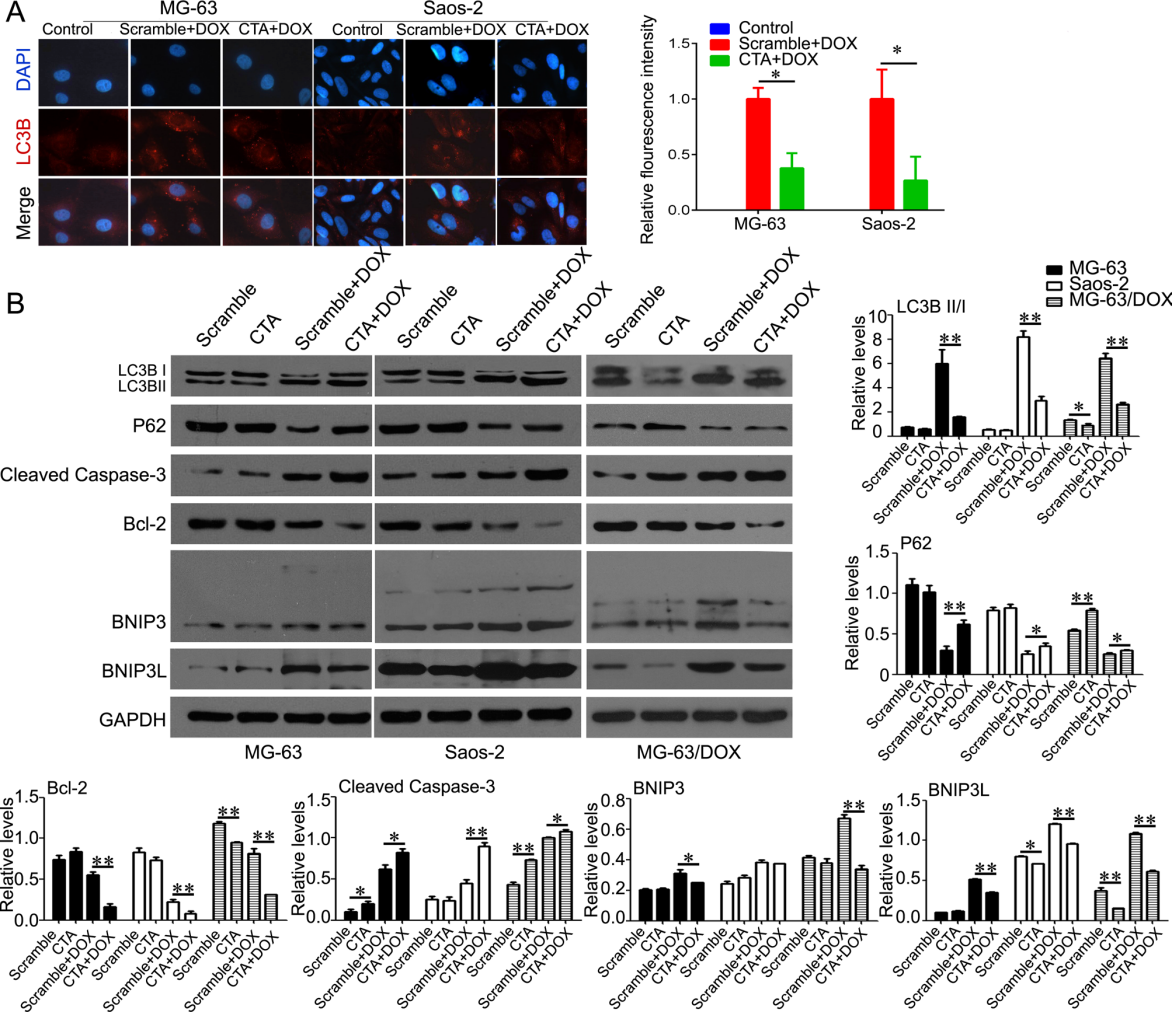
LncRNA CTA inhibits autophagy that induced by DOX (**A**) Immunofluorescence was performed to detect the expression of LC3B and its location (left), and quantification of the red fluorescence intensity (right). (**B**) Western blot was performed to analyze the expression of the key molecules of autophagy signaling, including LC3BI, LC3BII, P62, cleaved caspase 3, Bcl-2, BNIP3 and BNIP3L, and quantification. All the experiments were independently performed three times. Data were presented as mean ± standard deviation. *P < 0.05, **P < 0.01.

Previous study showed that activation of autophagy is a cytoprotective event to limit proteasome inhibitor-induced cell death [23]. Here, we evaluated whether overexpression of lncRNA CTA would trigger inhibition of autophagy to overcome the DOX resistance in osteosarcoma cells. MG63, Saos-2 and MG63/DOX cells were transfected with the lentivirus expressed lncRNA CTA and scramble control (control), and then the cells were treated with or without DOX (0.1 μM) for 24 hours. Onset of autophagy was visualized by immunofluorescence microscopy. As shown in Figure 5A, DOX stimulation induced a punctate LC3B localization characteristic of autophagosome formation and increased the fluorescence intensity in MG63 and Saos-2 cells, indicating that autophagy was activated. And overexpression of lncRNA CTA in MG63 and Saos-2 cells could decrease the punctate accumulation and the fluorescence intensity of LC3B in cytoplasm mediated by DOX stimulation. Autophagy is a vacuolar process of cytoplasmic degradation by lysosome. Autophagy activation or inactivation following DOX stimulation and lncRNA CTA overexpression was further confirmed by measuring protein expression levels of the LC3-I and LC3-II isoforms by western blot analysis. Here, we found that MG63, Saos-2 and MG63/DOX cells treated with DOX had increased accumulation of LC3-II isoforms as well as upregulation of BNIP3/BNIP3L expression, whereas lncRNA CTA overexpression reversed this accumulation of LC3-II isoforms and upregulation of BNIP3/BNIP3L expression in MG63, Saos-2 and MG63/DOX. In addition, we also observed that lncRNA CTA exerted a synergic effect with DOX in increasing the levels of Caspase 3 and in decreasing the levels of Bcl-2 (Figure 5B).

### LncRNA CTA exerts a synergic effect with DOX in growth inhibition of osteosarcoma cells in nude mice

**Figure 6:**
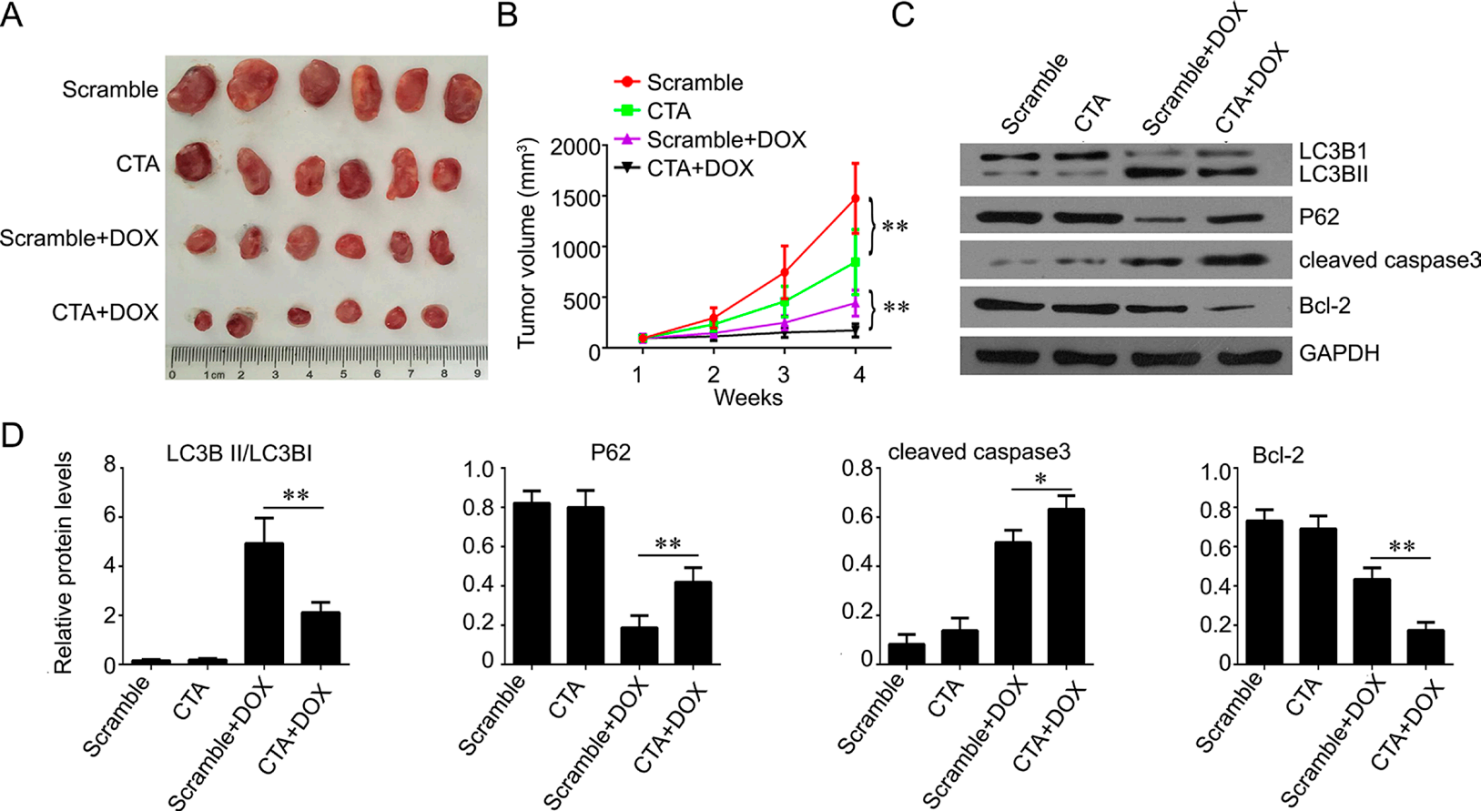
LncRNA CTA inhibits osteosarcoma growth *in vivo* (**A**) On 4 weeks after implantation, the nude mice in each group were sacrificed, and the xenografted osteosarcoma was obtained. (**B**) The tumor volume was calculated. (**C**) Western blot was performed to analyze the expression of the key molecules of autophagy signaling, including LC3B1, LC3BII, P62, cleaved caspase 3 and Bcl-2. (**D**) Quantification of the band of western blot in (C). Data were presented as mean ± standard deviation. *P < 0.05, **P < 0.01.

Furthermore, we studied the function of lncRNA CTA in the growth of xenografted osteosarcoma *in vivo*. LncRNA CTA expressed lentivirus were used to infect MG-63 cells. These MG63 cells were then subcutaneously implanted into nude mice. One week after implantation, the mice were injected with DOX (15 mg/kg) by tail vein for three weeks. We found that lncRNA CTA overexpression in MG63 cells slightly inhibited the tumor growth. However, lncRNA CTA overexpression significantly enhanced DOX-induced growth inhibition in nude mice, which was evaluated by decrease tumor volume (Figure 6A–6B). On 4 weeks after implantation, all mice were sacrificed by injection with overdose chloral hydrate. The tumor tissues were obtained and used for western blot analysis. Consistent with the results *in vitro*, we found that lncRNA CTA overexpression decreased the accumulation of LC3-II isoforms in tumor tissues (Figure 6C–6D), and exerted a synergic effect with DOX in increasing the levels of caspase 3 and in decreasing the levels of Bcl-2.

### LncRNA CTA is a direct target of miR-210 in osteosarcoma cells

**Figure 7:**
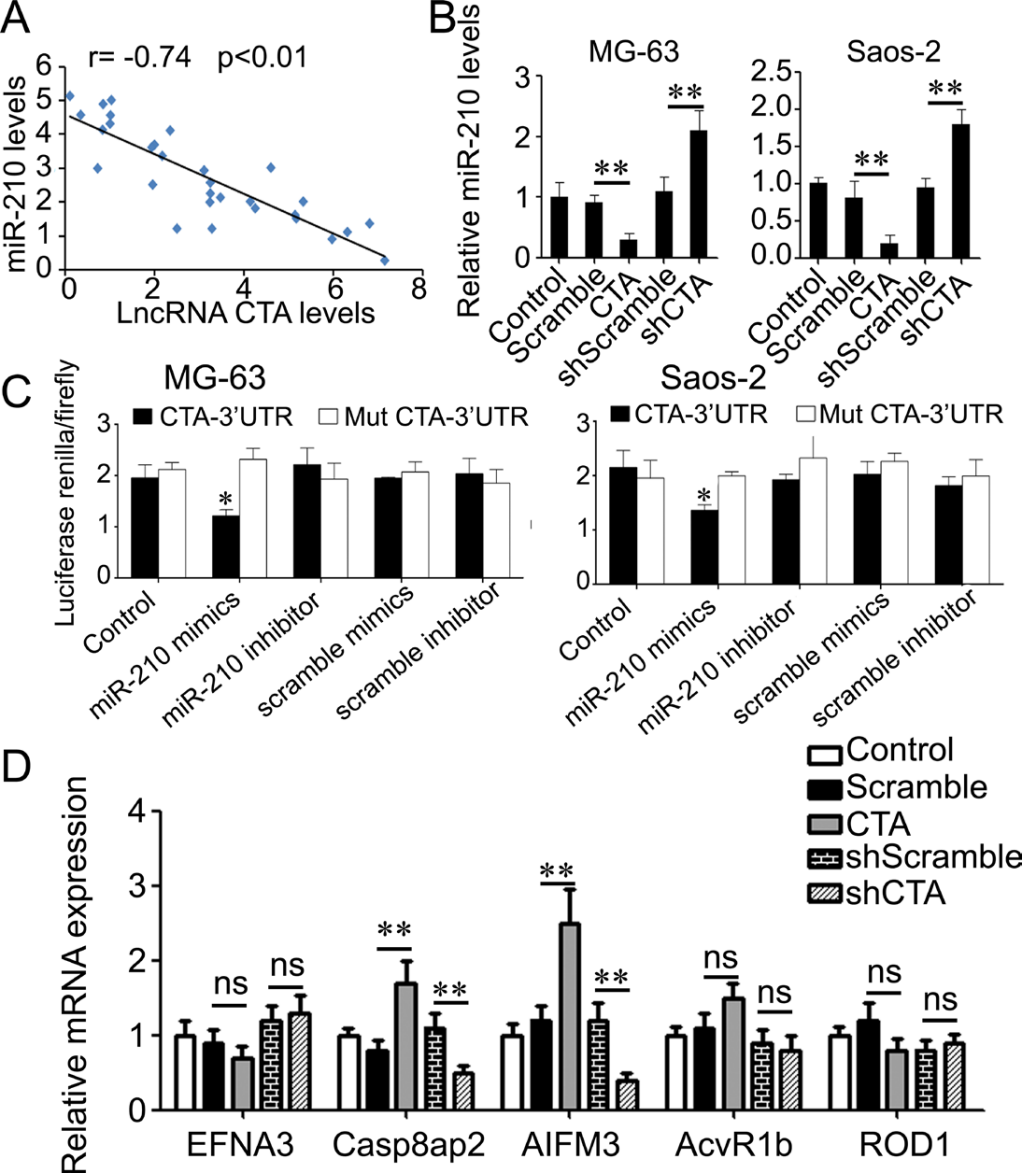
LncRNA CTA regulates miR-210′s targets by competitively binding to miR-210 (**A**) LncRNA CTA was inversely correlative with miR-210 in osteosarcoma tissues. (**B**) Real-time RT-PCR was performed to determine the relative miR-210 level in osteosarcoma MG-63 and Saos-2 cells transfected with lncRNA CTA, CTA-shRNA or scramble control. (**C**) The luciferase activity was notably decreased in osteosarcoma MG-63 and Saos-2 cells co-transfected with miR-210 mimics and CTA 3′UTR, but unaltered in osteosarcoma MG-63 and Saos-2 cells co-transfected with miR-210 mimics and Mut CTA 3′UTR. (**D**) Real-time RT-PCR was performed to determine the relative mRNA level of miR-210 targets in osteosarcoma MG-63 and Saos-2 cells transfected with lncRNA CTA, CTA-shRNA or scramble control. Data were presented as mean ± standard deviation. ns, no significance.*P < 0.05, **P < 0.01.

Finally, we analyzed the correlation between lncRNA CTA and miR-210 expression in 30 cases of osteosarcoma tissues. We found that there was a negative correlation between lncRNA CTA and miR-210 expression in osteosarcoma tissues (*r* = −0.74, *p* < 0.01, Figure 7A). We also found that the levels of miR-210 were markedly reduced in lncRNA CTA-overexpressing MG63 and Saos-2 cells, whereas significantly increased in lncRNA CTA-downregulating MG63 and Saos-2 cells (Figure 7B).

Bioinformatic analysis showed that lncRNA CTA was a direct target of miR-210. The WT- lncRNA CTA -3′UTR or Mut-lncRNA CTA -3′UTR luciferase reporter vector was generated, respectively. Therefore, we further performed luciferase reporter assay to confirm their relationship in MG63 and Saos-2 cells. As shown in Figure 7C, The MG63 and Saos-2 cells co-transfected with miR-210 mimics and WT- lncRNA CTA -3′UTR vector exhibited significantly downregulated the luciferase activity, which was eliminated by co-transfection with miR-210 mimic and Mut- lncRNA CTA -3′UTR vector, indicating that miR-210 could directly bind to the 3′UTR of lncRNA CTA. In addition, we also analyzed the expression of potential targets of miR-210 in MG63 and Saos-2 cells transfected with lncRNA CTA and scramble control. We found that overexpression of lncRNA CTA significantly upregulated the expression of Casp8ap2 and AIFM3, but did not alter the expression of EFNA3, AcvR1b and ROD1, while knockdown of CTA downregulated the expression of Casp8ap2 and AIFM3, and yet did not change the expression of EFNA3, AcvR1b and ROD1 (Figure 7D).

## DISCUSSION

In this study, we found that lncRNA CTA could be activated by DOX. On the other hand, lncRNA CTA was downregulated in DOX-resistant cells. Upregulation of lncRNA CTA promoted apoptosis by competitively binding miR-210 and inhibiting autophagy in osteosarcoma cells.

Due to chemoresistance, the prognosis of osteosarcoma so far is still little optimism. Increasing studies have shown that lncRNAs play an important role in tumorigenesis and chemoresistance [24, 25], but little is known about the DOX-induced lncRNAs in osteosarcoma cells. In the present study, we screened for miR-210-related and DOX-altered lncRNAs using bioinformatic tool analysis, which were further confirmed by using qPCR. We observed that lncRNA CTA was markedly decreased in osteosarcoma tissues, and low expression of lncRNA CTA predicted a poor prognosis of patients with osteosarcoma. In addition, we found that lncRNA CTA was significantly increased by DOX in MG63 cells. We also found that lncRNA CTA was downregulated in DOX-resistant cells and this decrease contributed to chemoresistance. The molecular mechanism by which DOX treatment alters the levels of lncRNA CTA remains unknown and will be investigated in future. Subsequently, to discover pathway regulated by lncRNA CTA in response to DOX treatment, we found that inactivation of autophagy was associated with lncRNA CTA. DOX has been widely used in treatment of osteosarcoma [26]. Zhao D et al found that Dox treatment induced increased expression levels of cleaved caspase-3 and LC3, whereas reduced those of p62 in osteosarcoma cells; and inhibition of autophagy notably enhanced the effects of DOX [27]. In this study, we also found that the autophagy activity was significantly increased responding to DOX stimulation, while overexpression of lncRNA CTA reduced autophagy activity and promoted chemotherapy-induced apoptosis *in vitro* and *in vivo*, which may be involved in decrease of Bcl-2 expression. Baranski Z et al demonstrated that pharmacological inhibition of a range of Bcl-2 family members exerted a synergistic effect on DOX-mediated apoptosis [28].

Accumulating evidence suggest that lncRNAs act as miR sponges and inhibit miR functions [29]. Recently, the lncRNA associated ceRNA networks were revealed to play an important role in many types of human cancer, including ovarian cancer, breast carcinoma, and glioma [30–32]. For example, Wang Y demonstrated that lncRNA LINC00161 could bind miR-645, which was reported to target IFIT2, and regulated IFIT2 expression via miR-645; and LINC00161 sensitized osteosarcoma cells to cisplatin-induced apoptosis through regulation of the miR-645-IFIT2 axis [33]. We here found there was an inverse relationship between lncRNA CTA and miR-210 expression in OS tissues. We also confirmed that lncRNA CTA harbored a miR-210 binding site, and their interaction resulted in suppression of miR-210. Recently, increasing evidences have confirmed that miR-210 functions as an oncogene in many types of cancers by degrading its targets such as E2F3, Casp8ap2 and ATM [34–36]. Here, we showed that induced lncRNA CTA resulted in significant increase of Caspase-8-associated Protein 2 (Casp8ap2) and apoptosis-inducing factor, mitochondrion-associated 3 (AIFM3), two valid targets of miR-210 [37, 38]. It is therefore reasonable that the lncRNA CTA -mediated growth inhibition is at least in part due to the specific suppression of miR-210 in osteosarcoma cells. In addition, we should note that CTA may also target to other miRNAs except to miR-210, and therefore exhibits different functions. On the other hand, we here found that overexpression of lncRNA CTA significantly upregulated the expression of targets of miR-210, Casp8ap2 and AIFM3, but did not alter the expression of EFNA3, AcvR1b and ROD1 (revised Figure 7D), indicating that CTA may directly suppress tumor growth by regulating these proteins. However, miR-210 also target to Bcl-2 adenovirus E1B 19kDa-interacting protein 3 (BNIP3) [39], vacuole membrane protein 1 (VMP1) [40] and fibroblast growth factor receptor-like 1 (FGFRL1) [41]. Whether CTA regulates these genes expression remains to be investigated. And we cannot exclude the other unknown pathways that would participate to role of CTA in tumor development.

In conclusion, these findings demonstrate that lncRNA CTA is an essential regulator in DOX-induced apoptosis, and the lncRNA CTA/miR-210 signaling axis plays an important role in overcoming osteosarcoma chemoresistance. Therefore, our study provides a potential therapeutic strategy for osteosarcoma treatment.

## MATERIALS AND METHODS

### Patients and samples collection

This study was approved by the Ethical Committee of the Third Xiangya Hospital of Central South University, P.R. China. Informed consents were obtained from all patients involved in this study. The osteosarcoma tissues and their matched adjacent non-tumor tissues were collected from 30 cases of osteosarcoma patients when surgical resection at the Third Xiangya Hospital between March 2005 and Oct 2010, and were confirmed by histopathological evaluation. All osteosarcoma patients received no preoperative radiotherapy and/or chemotherapy. The collected tissues were immediately stored at −80°C until use.

### Cell lines and cell culture

Three osteosarcoma cell lines Saos-2, U-2OS and MG-63 and a normal human osteoblast were purchased from Cell bank of Chinese Academy of Sciences, Shanghai, China. And the DOX (DOX) resistant cell lines MG-63/DOX were purchased from ATCC Corporation (Gaithersburg, MD, USA). All cells were cultured in DMEM (Life Technologies, Carlsbad, CA, USA) supplemented with 10% FBS (Life Technologies) at 37°C with 5% CO_2_.

### RNA extraction and quantitative real-time reverse transcription PCR

Total RNA was isolated from osteosarcoma and matched adjacent tissues or the indicated cells by using Trizol Reagent (Life Technologies), according to the manufacturer's protocol. The concentration and purity of total RNA were measured on a Nanodrop spectrophotometer (Thermo Scientific, Waltham, MA, USA). The total RNA (10 ng) was then converted into cDNA by using a PrimeScript 1st Strand cDNA Synthesis Kit (TAKARA, Dalian, China), according to the manufacturer's protocol. After that, quantitative real-time PCR was conducted to detect the expression of lncRNAs and miR-210 using SYBR-Green PCR kit (Takara), according to the manufacturer's protocol. The reaction conditions was 95°C for 5 min, followed by 40 cycles of 95°C for 15 sec and 60°C for 30 sec. GAPDH and U6 was used as an internal control for lncRNAs and miR-210, respectively. All of the PCR primers were obtained from Life Technologies. The primer for miR-210, and U6 were purchased from (GeneCopoeia Inc. USA).The other primers used in this study were shown as Table 2. The relative expression level was determined using the 2^−ΔΔCt^ method.

### *In situ* hybridization in tissue microarray

The tissue microarray containing 92 cases of osteosarcoma tissues (Auragene Co Ltd., Changsha, China) and 12 cases of normal bone tissues (BIOMAX, Xian, China) were purchased. The 92 osteosarcoma patients had valid follow-up data. The overall survival (OS) was defined as the time from diagnosis to the date of death or the date last known alive. Clinicopathologic characteristics of patients were recorded including gender, age, clinical tumor node-metastasis (TNM) stage, tumor size and pathology diagnosis. The profile of clinicopathologic characteristics of the osteosarcoma patients was shown in Table 2. This study was approved by the Committee on the Ethics of the Third Xiangya Hospital of Central South University. All of the individuals participating or their dependents have written informed consent. The ISH probe used for detecting ENSG00000253603.1 (lncRNA CTA) labeled Digoxin was designed and synthesized by Life Technologies (Shanghai, China). Slices were processed using Enhanced Sensitive ISH Detection Kit I (POD) (cat: MK1030, Boster, Wuhan, China) according to manufacturer's protocol. The slides were stained and counterstained with haematoxylin for 90 seconds. The slides were mounted and dried. Slides were photographed with Olympus BX51 microscope (Olympus, Japan). The positive signals were shown in blue color.

The slides were evaluated by two independent pathologists under a light microscope (BX51, Olympus, Japan). LncRNA CTA staining intensity was scored as 0 (negative, -), 1 (weak, +), 2 (moderate, ++), and 3 (strong, +++). The extent of staining was scored as 0~1.0 (0%~100%). The final staining score (0–3) was calculated as the multiplication of the intensity score and extent score. The expression of lncRNA CTA was scored as high expression (≥ 1) or low expression (< 1). To compare the expression of lncRNA CTA between normal and tumor tissues, the score of lncRNA CTA expression was normalized to the average score of lncRNA CTA in normal tissues.

### Western blotting

Cells were lysed in cold RIPA buffer, and the protein was separated with 12% SDS-PAGE, which was then transferred to PVDF membrane (Thermo Fisher). After that, the membrane was incubated in PBS with 5% nonfat dried milk (Yili, Beijing, China) for 3 hr at 4°C. Then, the membrane was incubated with primary antibodies (mouse monoclonal anti-LC3BI/II, rabbit polyclonal anti-P62, mouse monoclonal anti-caspase 3, mouse monoclonal anti-Bcl-2, rabbit polyclonal anti-BNIP3, rabbit polyclonal anti-BNIP3L and mouse monoclonal anti-GAPDH, from Abcam, Cambridge, MA, USA) overnight at 4°C, and then with appropriate secondary antibody (Abcam) for 1 hr at 37°C. The immune complexes were detected using ECL Western Blotting Kit (Thermo Fisher). The relative protein expression was analyzed using Image-Pro plus software 6.0, and GAPDH was used as the internal reference.

### Cell transfection

Cell transfection was conducted using Lipofectamine 3000 (Life Technologies), according to the manufacturer's instruction. All of the plasmids and vectors used in this study were designed and purchased from RiboBio (Guangzhou RiboBio Co., Ltd., Guangzhou, China). In brief, serum-free medium was used to dilute scramble lncRNA, lncRNA CTA, scramble miR mimics, scramble miR inhibitor, miR-210 mimics and miR-210 inhibitors, which was then added with the diluted Lipofectamine 3000, and incubated at room temperature for 20 min. After that, they were added into the cell suspension. After incubated for 6 h, the medium was replaced by DMEM with 10% FBS. After transfection for 48 h, the cells were used for further analysis.

### Dual luciferase reporter assay

For determine the target relationship between miR-210 and lncRNA CTA, we generated the wild type (WT) and mutant type (MT) 3′-UTR of lncRNA CTA, which was then inserted into the MCS of the psiCHECK TM2 luciferase reporter vector, respectively. MG-63 and Saos-2 cells were transfected with WT- lncRNA CTA -3′UTR or MT- lncRNA CTA -3′UTR vector, plus scramble mimics/miR-210 mimics, or scramble inhibitor/miR-210 inhibitor, respectively. At the 48 hr after transfection, the renilla luciferase activity and firefly luciferase activity were determined using the dual-luciferase reporter assay system. Renilla luciferase activity was normalized to firefly luciferase activity.

### MTT assay

MG-63 and Saos-2 cells transfected with scramble or lncRNA CTA, and the blank control cells were seeded in 96-well plate for 12 hr. And the cells were treated with DOX (0.001, 0.01, 0.1, 1 μM) for 24 hr. After that, 100 μl of DMEM containing 5 mg/ml MTT were added into the wells, and incubated 37°C for 4 h. The MTT medium was removed, and 50 μl of DMSO was added, and incubated at 37°C for 15 min. The optical density (OD) at 570 nm was measured using the ELx800 Absorbance Microplate Reader (Biotek, USA). The experiments were repeated three times. The inhibition rate was calculated by using the equation:

(OD of scramble group – OD of CTA group)/ OD of scramble group×100%.

### Stable transfection and tumor growth analysis

Male BALB/C-nu/nu nude mice (4 weeks) were maintained at the Animal Center of our hospital. The pYr-LVX- lncRNA CTA lentiviral plasmid was obtained from Auragene Co Ltd., Changsha, China. MG63 cells were then stably transfected with pYr-LVX- lncRNA CTA lentiviral plasmid. Blank pLVX-IRES-ZsGreen1 vector was used as control. To determine the effect of lncRNA CTA on the tumorigenesis of osteosarcoma cells *in vivo*, nude mice (*n* = 6) were injected subcutaneously in the dorsal flank with 5 × 10^6^ MG63 cells stably transfected with lncRNA CTA -overexpressing plasmid; and the control group (*n* = 6) were injected with MG63 cells stably transfected with the scramble vector. Additionally, to determine the synergic effect of lncRNA CTA and DOX on the tumorigenesis of osteosarcoma cells *in vivo*, one week after cells injection, the nude mice were injected with DOX (15 mg/kg) by tail vein for twice every week, and saline injection was used as control. The tumor volumes were measured every week. Tumor volume was calculated by using the formula V (mm3) = 0.2618 × a× b ×(a + b) (a, maximum length to diameter; b, maximum transverse diameter). Four weeks after treatment, the mice were sacrificed by injection with overdose chloral hydrate. And the tumor tissues were used for western blot analysis.

### Immunofluorescence staining

The cells were smeared on slices and fixed with 4% paraformaldehyde. The slices were then blocked with 5% normal goat serum, and incubated with primary antibody mouse monoclonal anti-LC3B antibody (1:200 dilution) overnight at 4°C. The slices were then washed with TBST and incubated with Alexa Fluor 488-conjugated goat anti-mouse antibody for 60 min at 37°C. Then the slices were washed with TBST and counterstained with DAPI (1:1000 dilution; Sigma) for 5 min. The samples were visualized under a microscope (Nikon) through the program DP2-BSW.

### Flow cytometric analysis of the cell apoptosis

Following indicated treatment, the cells were trypsinized and washed with ice-cold PBS. The cell suspensions were incubated for 15 min in dark by using Annexin V/PI detection kit (Molecular Probes Inc., Eugene, OR) according to the manufacturer's instructions. And then, the cell apoptosis was analyzed by flow cytometry (Beckman Coulter, USA). The experiments were performed in triplicate.

### Terminal deoxynucleotidyl transferase (TdT) dUTP Nick-End Labeling (TUNEL) Assay

MG-63 and Saos-2 osteosarcoma cells were plated in chambered slides and transfected with scramble lncRNA or lncRNA CTA. And then, the cells were treated with or without DOX (0.1 μM) for 24 hr. After that, TUNEL assay was carried out using DeadEnd™ Colorimetric TUNEL System from Promega following manufacturer instruction. Images of cells were taken using Olympus IX71 microscope at a magnification of 40×. Images were analyzed and quantified using ImageJ.

### Statistical analysis

In this study, all experiments were repeated at least three times, and all data are expressed as the mean ± standard deviation. SPSS 18.0 software package (SPSS, Chicago, IL, USA) was used to perform statistical analysis. Difference between two groups was compared by independent-samples *t* test. Difference among three or more groups was compared by One-Way ANOVA. Chi-square test was applied to determine the association between the lncRNA CTA expression and the clinicopathological parameter of osteosarcoma. The Kaplan-Meier method and log-rank test were used to evaluate and compare the prognosis of osteosarcoma patient. The relationship between lncRNA CTA and miR-210 expression was determined by Pearson correlation analysis. The *P* value less than 0.05 were considered statistically significant.

## Abbreviations

OS: osteosarcoma
lncRNA: long non-coding RNA
MiR: microRNA
TUNEL: terminal deoxynucleotidyl transferase (TdT)
dUTP: Nick-End Labeling
RT-PCR: reverse transcription-polymerase chain reaction

## References

[1] Hattinger CM, Fanelli M, Tavanti E, Vella S, Ferrari S, Picci P, Serra M (2015). Advances in emerging drugs for osteosarcoma. Expert Opin Emerg Drugs.

[2] Yang Z, Li X, Yang Y, He Z, Qu X, Zhang Y (2016). Long noncoding RNAs in the progression, metastasis, and prognosis of osteosarcoma. Cell Death Dis.

[3] Chang L, Shrestha S, LaChaud G, Scott MA, James AW (2015). Review of microRNA in osteosarcoma and chondrosarcoma. Med Oncol.

[4] Ying S, Jianjun H, Xue Y, Shuwei Y, Liyuan Z, Jie W, Lixian C (2017). MicroRNA-133b inhibits cell proliferation and invasion in osteosarcoma by targeting to Sirt1. Oncol Res.

[5] Liu W, Xiao P, Wu H, Wang L, Kong D, Yu F (2016). MicroRNA-98 plays a suppressive role in non-small cell lung cancer through inhibition of SALL4 protein expression. Oncol Res.

[6] Liang T, Hu XY, Li YH, Tian BQ, Li ZW, Fu Q (2016). MicroRNA-21 Regulates the Proliferation, Differentiation, and Apoptosis of Human Renal Cell Carcinoma Cells by the mTOR-STAT3 Signaling Pathway. Oncol Res.

[7] Miao J, Wu S, Peng Z, Tania M, Zhang C (2013). MicroRNAs in osteosarcoma: diagnostic and therapeutic aspects. Tumour Biol.

[8] Irlam-Jones JJ, Eustace A, Denley H, Choudhury A, Harris AL, Hoskin PJ, West CM (2016). Expression of miR-210 in relation to other measures of hypoxia and prediction of benefit from hypoxia modification in patients with bladder cancer. Br J Cancer.

[9] Kai AK, Chan LK, Lo RC, Lee JM, Wong CC, Wong JC, Ng IO (2016). Down-regulation of TIMP2 by HIF-1alpha/miR-210/HIF-3alpha regulatory feedback circuit enhances cancer metastasis in hepatocellular carcinoma. Hepatology.

[10] Xie X, Wu W, Liang L, Han S, Chen T, Pan S, Xue M, Li S (2015). Prognostic role of microRNA-210 in various carcinomas: a meta-analysis. Int J Clin Exp Med.

[11] Wang Z, Yin B, Wang B, Ma Z, Liu W, Lv G (2013). MicroRNA-210 promotes proliferation and invasion of peripheral nerve sheath tumor cells targeting EFNA3. Oncol Res.

[12] Puissegur MP, Mazure NM, Bertero T, Pradelli L, Grosso S, Robbe-Sermesant K, Maurin T, Lebrigand K, Cardinaud B, Hofman V, Fourre S, Magnone V, Ricci JE (2011). miR-210 is overexpressed in late stages of lung cancer and mediates mitochondrial alterations associated with modulation of HIF-1 activity. Cell Death Differ.

[13] Wang J, Raimondo M, Guha S, Chen J, Diao L, Dong X, Wallace MB, Killary AM, Frazier ML, Woodward TA, Wang J, Sen S (2014). Circulating microRNAs in Pancreatic Juice as Candidate Biomarkers of Pancreatic Cancer. J Cancer.

[14] Lai NS, Wu DG, Fang XG, Lin YC, Chen SS, Li ZB, Xu SS (2015). Serum microRNA-210 as a potential noninvasive biomarker for the diagnosis and prognosis of glioma. Br J Cancer.

[15] Liu D, Xia H, Wang F, Chen C, Long J (2016). MicroRNA-210 interacts with FBXO31 to regulate cancer proliferation cell cycle and migration in human breast cancer. Onco Targets Ther.

[16] Cai H, Lin L, Cai H, Tang M, Wang Z (2013). Prognostic evaluation of microRNA-210 expression in pediatric osteosarcoma. Med Oncol.

[17] He X, Sun G, Guo F, Wang K, Gao Y, Feng Y, Song B, Li W, Li Y (2016). Knockdown of long non-coding RNA FTX inhibits proliferation, migration, and invasion in renal cell carcinoma cells. Oncol Res.

[18] Zhou J, Li X, Wu M, Lin C, Guo Y, Tian B (2016). Knockdown of Long Noncoding RNA GHET1 Inhibits Cell Proliferation and Invasion of Colorectal Cancer. Oncol Res.

[19] Li J, Meng H, Bai Y, Wang K (2016). Regulation of lncRNA and Its Role in Cancer Metastasis. Oncol Res.

[20] Zhao H, Hou W, Tao J, Zhao Y, Wan G, Ma C, Xu H (2016). Upregulation of lncRNA HNF1A-AS1 promotes cell proliferation and metastasis in osteosarcoma through activation of the Wnt/beta-catenin signaling pathway. Am J Transl Res.

[21] Li W, Xie P, Ruan WH (2016). Overexpression of lncRNA UCA1 promotes osteosarcoma progression and correlates with poor prognosis. J Bone Oncol.

[22] Wang Y, Zhang L, Zheng X, Zhong W, Tian X, Yin B, Tian K, Zhang W (2016). Long non-coding RNA LINC00161 sensitises osteosarcoma cells to cisplatin-induced apoptosis by regulating the miR-645-IFIT2 axis. Cancer Lett.

[23] Hollomon MG, Gordon N, Santiago-O’Farrill JM, Kleinerman ES (2013). Knockdown of autophagy-related protein 5, ATG5, decreases oxidative stress and has an opposing effect on camptothecin-induced cytotoxicity in osteosarcoma cells. Bmc Cancer.

[24] Wang Y, Zhang L, Zheng X, Zhong W, Tian X, Yin B, Tian K, Zhang W (2016). Long non-coding RNA LINC00161 sensitises osteosarcoma cells to cisplatin-induced apoptosis by regulating the miR-645-IFIT2 axis. Cancer Lett.

[25] Li Z, Zhao L, Wang Q (2016). Overexpression of long non-coding RNA HOTTIP increases chemoresistance of osteosarcoma cell by activating the Wnt/beta-catenin pathway. Am J Transl Res.

[26] Ji P, Yu L, Guo WC, Mei HJ, Wang XJ, Chen H, Fang S, Yang J (2014). Doxorubicin Inhibits Proliferation of Osteosarcoma Cells Through Upregulation of the Notch Signaling Pathway. Oncol Res.

[27] Zhao D, Yuan H, Yi F, Meng C, Zhu Q (2014). Autophagy prevents doxorubicininduced apoptosis in osteosarcoma. Mol Med Rep.

[28] Baranski Z, de Jong Y, Ilkova T, Peterse EF, Cleton-Jansen AM, van de Water B, Hogendoorn PC, Bovee JV, Danen EH (2015). Pharmacological inhibition of Bcl-xL sensitizes osteosarcoma to doxorubicin. Oncotarget.

[29] Liu Q, Huang J, Zhou N, Zhang Z, Zhang A, Lu Z, Wu F, Mo YY (2013). LncRNA loc285194 is a p53-regulated tumor suppressor. Nucleic Acids Res.

[30] Zhang Z, Cheng J, Wu Y, Qiu J, Sun Y, Tong X (2016). LncRNA HOTAIR controls the expression of Rab22a by sponging miR-373 in ovarian cancer. Mol Med Rep.

[31] Ma CC, Xiong Z, Zhu GN, Wang C, Zong G, Wang HL, Bian EB, Zhao B (2016). Long non-coding RNA ATB promotes glioma malignancy by negatively regulating miR-200a. J Exp Clin Cancer Res.

[32] Yang C, Wu D, Gao L, Liu X, Jin Y, Wang D, Wang T, Li X (2016). Competing endogenous RNA networks in human cancer: hypothesis, validation, and perspectives. Oncotarget.

[33] Wang Y, Zhang L, Zheng X, Zhong W, Tian X, Yin B, Tian K, Zhang W (2016). Long non-coding RNA LINC00161 sensitises osteosarcoma cells to cisplatin-induced apoptosis by regulating the miR-645-IFIT2 axis. Cancer Lett.

[34] Dang K, Myers KA (2015). The role of hypoxia-induced miR-210 in cancer progression. Int J Mol Sci.

[35] Li M, Ma X, Li M, Zhang B, Huang J, Liu L, Wei Y (2014). Prognostic role of microRNA-210 in various carcinomas: a systematic review and meta-analysis. Dis Markers.

[36] Wang J, Zhao J, Shi M, Ding Y, Sun H, Yuan F, Zou Z (2014). Elevated expression of miR-210 predicts poor survival of cancer patients: a systematic review and meta-analysis. Plos One.

[37] Yang W, Sun T, Cao J, Liu F, Tian Y, Zhu W (2012). Downregulation of miR-210 expression inhibits proliferation, induces apoptosis and enhances radiosensitivity in hypoxic human hepatoma cells *in vitro*. Exp Cell Res.

[38] Kim HW, Haider HK, Jiang S, Ashraf M (2009). Ischemic preconditioning augments survival of stem cells via miR-210 expression by targeting caspase-8-associated protein 2. J Biol Chem.

[39] Wang F, Xiong L, Huang X, Zhao T, Wu LY, Liu ZH, Ding X, Liu S, Wu Y, Zhao Y, Wu K, Zhu LL, Fan M (2013). miR-210 suppresses BNIP3 to protect against the apoptosis of neural progenitor cells. Stem Cell Res.

[40] Ying Q, Liang L, Guo W, Zha R, Tian Q, Huang S, Yao J, Ding J, Bao M, Ge C, Yao M, Li J, He X (2011). Hypoxia-inducible microRNA-210 augments the metastatic potential of tumor cells by targeting vacuole membrane protein 1 in hepatocellular carcinoma. Hepatology.

[41] Tsuchiya S, Fujiwara T, Sato F, Shimada Y, Tanaka E, Sakai Y, Shimizu K, Tsujimoto G (2011). MicroRNA-210 regulates cancer cell proliferation through targeting fibroblast growth factor receptor-like 1 (FGFRL1). J Biol Chem.

